# Unmasking the Enigma: A Case Report of Catatonia Unveiled As Munchausen by Proxy

**DOI:** 10.7759/cureus.43082

**Published:** 2023-08-07

**Authors:** Anoop S Takher, Rosario M Cosme

**Affiliations:** 1 Child Psychiatry, Rush University Medical Center, Chicago, USA

**Keywords:** munchausen by proxy, child abuse, bfcrs, ativan challenge, pediatric catatonia

## Abstract

Catatonia is a behavioral syndrome characterized by a variety of symptoms such as mutism, stupor, rigidity, negativism, and verbigeration. It can be caused by various psychiatric and general medical conditions. While the diagnosis in the pediatric population is relatively uncommon, emerging literature supports a higher prevalence of catatonia in children. We present a 12-year-old girl with a complex medical and psychosocial history, including a functional neurological disorder and concerns for child abuse and Munchausen syndrome by proxy imposed by her mother. The patient was initially admitted for medical management of vomiting and refusal to eat. Child psychiatry was consulted for further assessment and noted multiple catatonic symptoms with a Busch-Francis catatonia rating scale (BFCRS) score of 22. A subsequent 1 mg IV lorazepam challenge test showed improvement in the patient's symptoms with a repeat BFCRS score of 10. This case supports emerging literature suggesting a higher prevalence of catatonia in children and the importance of recognizing this syndrome and its wide array of underlying causes.

## Introduction

Catatonia is a diagnostically challenging behavioral syndrome, and in children and adolescents, the most commonly seen features are mutism, immobility/stupor, staring, posturing, negativism, and rigidity [1]. Catatonia is generally considered difficult to diagnose due to the large variance in which it can present, and the frequency of catatonia in children is difficult to quantify with the limited literature available. It is estimated that catatonia in children may be more common than previously thought. However, pediatric catatonia is still underdiagnosed due to the difficulty of diagnosis in children with the preconceived notion that catatonia is rare and most commonly associated with psychiatric conditions such as schizophrenia, which tends to present in later adolescence or adulthood [2]. 

The diagnosis of catatonia can be challenging, but fortunately, there are diagnostic tools to aid treatment and diagnosis. The Bush-Francis Catatonia rating scale (BFCRS) is a useful 23-item scale (with each item scored from 0 to 3) used to standardize the screening for and quantify the symptom severity of catatonia [3]. Benzodiazepines are indicated not only as first-line treatment of catatonia but also as a diagnostic tool through a positive lorazepam challenge test (LCT), in which the patient has resolution of symptoms after administration of 1 to 2 mg of lorazepam [4]. Second-line treatment includes electroconvulsive therapy (ECT), and there have also been cases of alternative pharmacotherapies being utilized [5,6].

We present the case of a 12-year-old female with an extremely complex medical and psychosocial history who was seen by our child psychiatry consult liaison service. It was suspected that there was an underlying depression or anxiety, as well as suspected medical child abuse or factitious disorder imposed on another (more commonly referred to as Munchausen by proxy). After a careful physical exam, extensive chart review into medical records starting from birth, and scoring of BFCRS, a positive LCT was seen after induction of 1 mg IV lorazepam. A positive LCT is considered diagnostic for catatonia [4]. The patient’s progress was followed during her inpatient stay through subsequent visits and rescoring of the BFCRS.

## Case presentation

Our patient was a 12-year-old girl who was admitted from the ED accompanied by her mother for vomiting, regurgitation, headaches, abdominal pain, and refusal to eat for two days. The patient has a complex and extensive medical history with over 700 documented encounters within the healthcare system, including four separate child protective service (CPS) cases for concerns of child abuse and Munchausen by proxy imposed by the mother. She was seen by numerous different specialists with varying diagnoses, including but not limited to: functional neurologic disorder, common variable immune deficiency, pediatric autoimmune neuropsychiatric disorders associated with streptococcal (PANDAS) infections, partial IgA deficiency, attention-deficit/hyperactivity disorder (ADHD), dyslexia, migraines, congenital Lyme disease, hearing loss, gait abnormalities, unspecified pain, and avoidant restrictive food disorder. 

On arrival at general medicine, the patient had a full medical work-up. Her BMI was 14, and a past history of eating disorder symptoms was ruled out. The patient's kidney, ureter and bladder (KUB), ECG, vitals, and baseline labs were unremarkable. On examination, the patient appeared younger than stated, lying supine in bed with abnormal posturing and notable muscle wasting. Her upper extremities, hands, and wrists displayed flexion contractures, while the lower extremities were held flexed in a frog leg position. She displayed multiple signs indicating catatonia, including immobility/stupor, mutism, staring, posturing/catalepsy, verbigeration with hypernasality and slurred speech, rigidity, combativeness/excitement noted by aggressive and stereotypic speech, with clear signs of irritability, mutism, and withdrawal. An assessment of the patient by the BFCRS resulted in a rating of 22 out of a possible 66. The decision was made to initiate an LCT with 1 mg IV lorazepam. Within 15 minutes, the patient’s mutism, hypernasality, and slurring had improved remarkably, to the point where the patient carried on a conversation for the next hour. Additionally, her posture and rigidity improved, with her upper extremities able to achieve full extension, which had not been witnessed prior. Upon reassessment with the BFCRS, she was found to have a score of 10.

The patient was evaluated by pediatric CPS and subsequently separated from her parents and taken into protective custody through CPS, with all further healthcare decisions requiring approval through the Department of Children and Family Services (DCFS). The patient was transitioned to oral lorazepam with DCFS approval, starting at 0.25 mg 4 times daily with eventual titration up to 1.25 mg 4 times daily, which was tolerated well and resulted in significant improvement in mood and cognitive ability. Subsequent BFCRS fluctuated between 4 and 10 each day, with waning efficacy three to four hours after initial administration and alternating days of aggressive outbursts while the titration was increased to find optimal dosing. Zoloft was eventually started with titration up to 75 mg to target depressive and anxiety symptoms, which proved to be efficacious and resulted in an improvement in overall mood.

## Discussion

Although Catatonia can be caused by numerous psychiatric, neurologic, metabolic, and general medical conditions, it has been commonly mistaken for other conditions and often goes unconsidered as a diagnosis [7]. There has been increasing literature supporting a higher prevalence of catatonia in children, and this consideration is important to all pediatricians and psychiatrists, especially due to the shortage of child psychiatrists [8]. Studies indicate that schizophrenia and mood disorders are the two most commonly associated psychiatric disorders with catatonia [9]. It has also been found that catatonia has an incidence rate of 4% to 17% in adolescents and adults with autistic spectrum disorder and that childhood disintegrative disorders, Tourette's syndrome, Down's syndrome, and Prader-Willi syndrome are associated with higher rates of pediatric catatonia [8]. 

In addition to recognizing the relationship between catatonia and psychiatric disorders, it is important to realize the less commonly reported associations such as obsessive-compulsive disorder (OCD) [10] and group A Streptococcus which is not only associated with catatonia but can also cause pediatric acute-onset neuropsychiatric syndrome (PANS), which can present with OCD symptoms and severe food restriction [11]. There has also been literature implying adverse childhood events, including emotional or physical trauma and abuse, as a precipitating factor for catatonia in children. For example, there are studies from 1972 reporting a 10-year-old girl and an eight-year-old boy raised in poor familial circumstances with parental rejection who demonstrated negativistic and oppositional traits with symptoms of catatonia [1]. 

Our patient had multiple concerns for child abuse and Munchausen by proxy imposed by the mother, with four separate CPS cases opened in addition to the case opened during the current admission. It has been found that 97.6% of abusers are female, with 95.6% being the victim’s mother [12]. This case was particularly difficult to discern between true medical history and false impositions, but after thoroughly reviewing medical charts dating back to the patient’s birth, it was found that in addition to her history of medical child abuse, the patient has had positive group A Streptococcus results, a history of anxiety and OCD tendencies, and suspected autoimmune disfunction that apparently has responded to intravenous immunoglobulin (IVIG) in the past. Regardless, the patient had numerous risk factors and current symptomatology to warrant the investigation of catatonia through an LCT, which is not only diagnostic but also therapeutic. We witnessed a robust initial response to 1 mg ativan, with persistent relief while titrating up oral benzodiazepines throughout the hospital stay. Second-line treatment of catatonia includes ECT, which has been used successfully to treat numerous cases of catatonia in children with efficacy comparable to adult cases and has been shown to have a low risk of side effects including neuroleptic malignant syndrome (NMS) [13,14]. However, there is still resistance to the utilization of ECT in children, with some states requiring legal involvement to move forward with ECT. There has also been literature describing the utilization of alternative pharmacotherapies such as zolpidem [15] and even the atypical antipsychotic mirtazapine [4], which could also be helpful in targeting symptoms of weight loss as seen in a number of catatonia cases presenting with refusal to eat.

## Conclusions

This case further affirms the difficulties surrounding the identification, diagnosis, and treatment of catatonia in the pediatric population. Even in uncomplicated patients, catatonia is difficult to address, and it was even more so in the case of our patient with an extremely complex and complicated medical and social history. The long-term effects of an untreated or prolonged course of catatonia may also lead to life-threatening complications such as pulmonary embolism, aspiration pneumonia, and significantly increased mortality and morbidity. This case supports that although catatonia is more consistently found among specific disorders, including schizophrenia and autism spectrum disorder, other precipitating factors including adverse childhood events, anxiety, OCD, and other comorbidities must be considered when assessing pediatric patients for catatonia. Taking this into account, the diagnosis of catatonia with the LCT followed by first-line treatment of oral benzodiazepine or second-line ECT to target symptoms is warranted, as is treating the underlying disorder that may be contributing to precipitating the syndrome.
